# Is there a role for monocyte chemoattractant protein-1 in delirium? Novel observations in elderly hip fracture patients

**DOI:** 10.1186/s13104-015-1129-5

**Published:** 2015-05-06

**Authors:** Kjersti Skrede, Torgeir Bruun Wyller, Leiv Otto Watne, Ingebjørg Seljeflot, Vibeke Juliebø

**Affiliations:** Oslo Delirium Research Group, Oslo, Norway; Department of Geriatric Medicine, Oslo University Hospital, Oslo, Norway; Faculty of Medicine, University of Oslo, Oslo, Norway; Center for Clinical Heart Research, Oslo University Hospital, Oslo, Norway; Department of Cardiology, Oslo University Hospital, Oslo, Norway

**Keywords:** Delirium, Cytokines, Inflammation, Hip fracture

## Abstract

**Background:**

Delirium is common, associated with poor outcome, but its pathophysiology remains obscure. The aim of the present study was to study a possible role of monocyte chemoattractant protein-1 (MCP-1) in the development of delirium.

**Findings:**

A prospective cohort of 19 hip fracture patients (median age 83 years) were screened for delirium daily by validated methods. MCP-1 was measured on arrival and postoperatively. The number of patients with a raise in MCP-1 was statistically significantly higher in the group with delirium in the postoperative phase compared to the no-delirium group (5/6 vs. 1/7, p = .03).

**Conclusions:**

MCP-1 might play a role in the development of delirium.

## Background

Delirium (“acute confusional state”), defined as an acute and fluctuating change in consciousness and cognition, is a common condition associated with poor outcome [1]. Despite its common occurrence, the pathophysiology of delirium remains poorly understood. A number of mechanisms have been proposed, mainly emphasizing neurotransmission, inflammation, and chronic stress [2-4].

Monocyte chemoattractant protein-1 (MCP-1) is a chemokine that attracts leukocytes of the monocyte lineage [5]. Within the CNS, MCP-1 is produced mainly by astrocytes and resident microglia, and MCP-1 overexpression has been associated with neuroinflammatory conditions like multiple sclerosis, stroke and Alzheimer’s disease [6]. MCP-1 is constitutively expressed by both glial cells and neurons, suggesting a possible neuromodulating function [7] and has been shown to increase cell excitability and dopamine release in dopaminergic neurons in rat substantia nigra [8]. Since both neuroinflammation and neurotransmitter disturbances, especially acetylcholine deficiency and dopamine excess, have been proposed as pathophysiological mechanisms in delirium [9,10], we hypothesized a role of MCP-1 in the pathogenesis. To our knowledge, the role of MCP-1 in delirium has only been studied in ICU patients, showing a significant association between MCP-1 and delirium in patients with systemic inflammatory response syndrome (SIRS) [11].

The aim of the current study was thus to investigate the relationship between MCP-1, measured in serum preoperatively and postoperatively, and the development of delirium in a population of elderly hip fracture patients.

## Methods

This is a substudy of a prospective study designed to evaluate risk factors for preoperative and postoperative delirium in elderly hip fracture patients [12]. Patients aged ≥ 65, acutely admitted for a hip fracture, who spoke Norwegian, had no severe aphasia, head injury or terminal illness, and were admitted for at least 48 hours, were eligible. Patients admitted to Oslo University Hospital or Diakonhjemmet Hospital, Oslo, Norway, in the period from May through December 2006, providing a written informed consent and from whom at least one blood sample was drawn, were included in the current study.

The study was undertaken in accordance with the Declaration of Helsinki and approved by the Eastern Norway Regional Committee for Ethics in Medical Research (Project # 05075) and Ullevaal University Hospital Data Protection Officer. The patients were observed for a minimum of 48 hours and a maximum of 5 days after the operation.

Patients were screened for delirium within 48 hours of admission, thereafter on a daily basis (weekdays) until discharge or the fifth postoperative day. Delirium was diagnosed using the Confusion Assessment Method (CAM) criteria [13]: change in mental status with acute onset and/or fluctuating course, inattention, and disorganized thinking or altered level of consciousness. Details on the procedure have been published [12].

Pre-fracture cognitive function was determined using the short form of the Informant Questionnaire on Cognitive Decline in the Elderly (IQCODE), and patients with IQCODE scores 3.44 or greater were considered as probably suffering from pre-fracture cognitive decline [14]. The patients’ overall physical health was assessed according to the American Society of Anesthesiologists (ASA) score. The Barthel Index (maximum score 20) was scored by a close caregiver to give an indication of pre-fracture functioning in activities of daily living [15].

Blood samples were collected preoperatively and postoperatively, centrifuged at 3500 rpm for 10 minutes and stored at-70C. MCP-1 was measured by an enzyme-linked immunosorbent assay (ELISA) method with commercially available kits (R & D Systems Europe, Abingdon, Oxon, UK). To minimize run-to-run variability, serial samples from the same individuals were analyzed in the same microtiter run. In our laboratory the inter-assay coefficient of variation for MCP-1 was 9.2%.

Mann–Whitney U test was conducted to compare levels of MCP-1 markers in patients with and without delirium. For change in MCP-1 concentration, we had to dichotomize the variable (raise versus steady state or fall) and use the chi-square test as the distribution was not homoscedastic, and the conditions for using the Mann–Whitney U test thus not fulfilled [16]. For this analysis, every raise in the concentration (above zero) was defined as a raise.

## Findings

Preoperative blood samples were collected from 19 patients with median (interquartile range) age 83 (79–91) years (Table 1). 14 (74%) were women, 3 (16%) had ASA score >2, 5/16 (31%) had an IQCODE score > 3.44, and 5/16 (31%) had a premorbid Barthel Index score ≤ 18. All had at least three days of postoperative delirium assessments.

**Table 1:** **Participants in the study**

**Patient**	**Preoperative delirium**	**Postoperative delirium**	**Barthel**	**ASA**	**IQCODE**	**MCP1 preoperatively pg/mL**	**MCP1 postoperatively pg/mL**
1	Yes	Yes	8	3	5.00	274	-
2	No	No	20	2	2.94	308	-
3	No	Yes	19	2	4.31	298	-
4	No	No	20	2	3.00	430	-
5	No	Yes	-	2	-	515	-
6	No	No	20	2	3.06	334	-
7	Yes	Yes	16	3	4.88	879	424
8	Yes	Yes	16	2	4.81	237	318
9	No	No	19	2	3.06	372	350
10	Yes	Yes	19	2	3.12	356	509
11	Yes	Yes	-	2	-	255	521
12	No	No	20	2	3.00	303	260
13	-	Yes	18	2	4.81	255	521
14	-	No	20	2	3.06	334	485
15	-	Yes	20	2	3.13	265	673
16	-	No	20	3	3.00	384	334
17	-	No	20	2	3.00	284	274
18	-	No	17	2	3.00	778	650
19	-	No	-	2	-	473	329

Barthel: Barthel Activity of Daily Living Score. ASA: American Society of Anesthesiologists score. IQCODE: Informant Questionnaire on Cognitive Decline in the Elderly. MCP: Monocyte chemoattractant protein.

Twelve of the patients were screened for delirium preoperatively and 5 of them were found to be delirious at that time. There was no statistically significant difference in MCP-1 concentration between patients with and without preoperative delirium (median 274 and 334 pg/mL, respectively, p = 0.29, Mann–Whitney test).

**Figure 1:**
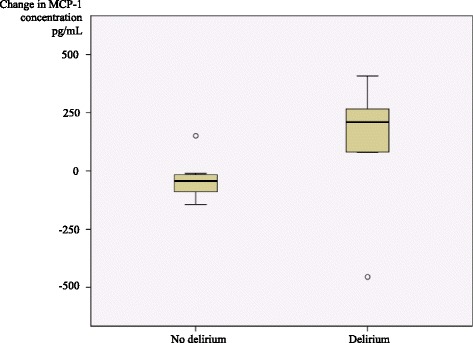
Boxplot of the change in serum concentration of monocyte chemoattractant protein-1 (MCP-1) from the preoperative to the postoperative phase by postoperative delirium status. n = 13.

In 13 of the 19 patients screened for delirium postoperatively, blood samples were collected both preoperatively and postoperatively (Table 1). Of these 13, 6 were delirious in the postoperative phase (regardless of time of onset), whereas 7 remained lucid throughout the stay. Five of 6 of patients with postoperative delirium and 1 of 7 without postoperative delirium had a rise in MCP-1 from the preoperative to the postoperative phase (p = 0.03, chi-square test). The median change from preoperative to postoperative measurement was 210 pg/mL in those with (n = 6), and-43 pg/mL in those without (n = 7) postoperative delirium (Figure 1).

## Discussion

A raise in MCP-1 was associated with postoperative delirium. The observation accords well with a hypothesis linking neuroinflammation, acetylcholine deficiency and dopamine excess to the pathophysiology of delirium [9].

Our findings are in line with the only other study that has measured MCP-1 in delirium [11]. That study also found that the serum levels of the pro-inflammatory cytokines IL-6 and IL-8 were higher in patients with delirium. The same cytokines have been reported to be higher in delirium also in other studies, both in serum [17,18] and in CSF [19]. A recently published study comprising 43 hip fracture patients found that CSF levels of IL-1ß was higher in patients with delirium, a finding that gives further support to a theory that inflammatory mechanisms are at play in the pathophysiology of delirium [20].

Support of increased inflammatory activity in the brain in delirium also come from a postmortem study were brain autopsies were performed within 24 hours after death in nine patients with delirium and six age-matched controls. In patients with delirium markers of microglial activity (HLA-DR and CD 68), astrocyte activity (GFAP) and IL-6 were increased [21].

Strengths of our study are its prospective design with comprehensive serial clinical evaluations, and the high sensitivity and specificity and low inter-assay coefficient of variation of the laboratory assay. Main limitations include the low number of patients, the fact that the time of collecting the blood samples was not standardized according to the time of surgery, and that the analyses are not accounting for diurnal or other physiological MCP-1 fluctuations in peripheral blood. The limited sample size provided insufficient power as to adjust for potential confounding factors by multivariate analysis. Since knowledge on delirium pathophysiology is rather scarce, we assume that the observation may be of interest, and give rise to more refined hypotheses that can be confirmed or rejected in larger studies.
